# Mosquito Saliva Subunit Vaccine Enhances Efficacy of VLP Vaccines Against Zika and Chikungunya Viruses

**DOI:** 10.3390/vaccines14080723

**Published:** 2026-08-21

**Authors:** Siân Jossi, Megan Cole, Yonca Keskek Turk, Sam Verwimp, Katrien Trappeniers, James Luk, Joshua X. D. Ang, Luke Alphey, Leen Delang, Clive S. McKimmie, Olga Pleguezuelos

**Affiliations:** 1 ConserV Bioscience Ltd., Heyford Park Innovation Centre, Upper Heyford, Bicester OX25 5HD, UKolga.pleguezuelos@immunity-consult.co.uk (O.P.); 2 Virus Host Interaction Team, Skin Research Centre, Hull York Medical School, University of York, York YO10 5DD, UK; yoncaturk@hacettepe.edu.tr; 3 Laboratory of Virus-Host Interactions and Therapeutic Applications, Department of Microbiology, Immunology and Transplantation, Rega Institute for Medical Research, Katholieke Universiteit (KU) Leuven, 3000 Leuven, Belgium; sam.verwimp@kuleuven.be (S.V.); katrien.trappeniers@kuleuven.be (K.T.); leen.delang@kuleuven.be (L.D.); 4 Department of Biology, University of York, York YO10 5DD, UK; yin.luk@york.ac.uk (J.L.); joshua.ang@york.ac.uk (J.X.D.A.); luke.alphey@york.ac.uk (L.A.); 5 York Biomedical Research Institute, University of York, York YO10 5DD, UK

**Keywords:** mosquito saliva, arbovirus, vaccine, Zika, chikungunya, peptides, VLPs, *Aedes*, *Culex*, T-cell, antibodies

## Abstract

**Background/Objectives**: Mosquito-borne viruses cause substantial global morbidity and mortality. Mosquito saliva, secreted during biting, facilitates blood feeding and can enhance virus infection. Here, we evaluated whether combining virus-specific antigens with mosquito salivary protein-derived peptides improves protection in Zika virus (ZIKV) and chikungunya virus (CHIKV) mouse models. **Methods**: Peptides from *Aedes* and *Culex* salivary proteins were selected based on conservation, predicted T-cell reactivity, and cellular and humoral immunogenicity in mice. Mice were immunised subcutaneously with a prime and boost two weeks apart using adjuvanted salivary peptides (SAL), commercial ZIKV- or CHIKV-like particles (VLPs), or salivary peptides combined with the relevant VLPs. Vaccine doses contained 0.5 µg VLPs and/or 10 nmol of each salivary peptide, formulated with Montanide ISA-51; when combined, VLPs and SAL were administered on opposite flanks. Two weeks after boosting, mice were challenged with ZIKV or CHIKV plus *Aedes aegypti* saliva. Challenge teams were blinded to group allocation until data analyses were complete. **Results**: SAL enhanced the protective effect of VLP vaccination compared with VLPs alone. In the CHIKV model, CHIKV VLP + SAL prevented joint swelling and reduced infectious virus in the inoculated ankle and viral RNA in the contralateral ankle. In the ZIKV model, ZIKV VLP + SAL reduced viral RNA in skin and brain tissue and improved survival. **Conclusions**: Mosquito salivary peptide antigens improved the protective efficacy of VLP vaccines against ZIKV and CHIKV in mouse models. Salivary antigens may provide a complementary vaccine component for multiple arboviruses transmitted by *Aedes* and *Culex* mosquitoes.

## 1. Introduction

Arboviruses are transmitted by arthropods, with mosquitoes representing the main vector for many human infections. Vector-borne diseases account for >17% of all infectious diseases, resulting in an estimated 700,000 deaths annually [1]. Mosquito-borne diseases specifically have a huge global economic and disease burden, and the annual number of infections is increasing due to the expansion of mosquito habitats through climate change, deforestation, and urbanisation [2]. Infections carried by *Aedes* mosquitoes alone were estimated to cost $95 billion in 2022, increasing 14-fold since 2013, with the burden disproportionately falling on low–middle income countries (LMICs) [3]. Female *Aedes* mosquitoes are the main vectors for the transmission of dengue, yellow fever, Zika virus (ZIKV), and chikungunya virus (CHIKV), whereas West Nile virus is transmitted primarily by *Culex* mosquitoes [1].

Outbreaks of ZIKV have been documented since 2007, with the most notable epidemic in 2015–2016 in South America where an estimated 707,133 autochthonous cases were reported across 48 countries [4]. ZIKV now persists endemically at lower levels [5] but still poses a major epidemic threat. Most ZIKV infections in adults are mild, but infection during pregnancy presents a significant health risk to the unborn child. ZIKV can cause severe congenital defects in the central nervous system of the foetus, resulting in developmental abnormalities and adverse pregnancy outcomes [6]. At the time of writing, there are no licenced vaccines or antiviral treatments for ZIKV.

In 2025 alone, a total of 445,271 cases and 155 deaths were attributed to CHIKV [7], including cases in Europe, highlighting the expansion of the geographic area affected by the virus beyond tropical climates into new geographic locations. CHIKV infections are characterized by acute onset of fever, rash and polyarthralgia, and symptoms can persist long-term. The consequences of these chronic CHIKV infections in particular carry a substantial economic and health burden, with an estimated annual loss of >106,000 Disability-Adjusted Life Years (DALYs) [8]. Two CHIKV vaccines are licensed for human use: the live-attenuated IXCHIQ (Valneva) licensed for use in adults aged 18 years and over, and the virus-like particle (VLP) Vimkunya (Bavarian Nordic) [9], licensed for use in individuals aged 12 years and over. Both vaccines were approved based on seroconversion data, with phase III efficacy data still pending, and limited safety data are available in pregnant women. Post-licensure use of IXCHIQ following CHIKV outbreaks has identified some safety concerns [10,11], with elderly people (>60) experiencing serious adverse events consistent with severe complications of chikungunya disease, including neurological and cardiac events. CHIKV vaccines suffer from a global inequity in access, with vaccines being available in high-income countries primarily for travellers, rather than being widely available to the populations of low- and middle-income countries (LMICs) most affected by the virus [12]. There are no antiviral treatments available, and protection of the population is largely dependent on mosquito control.

Current vaccine research largely focuses on eliciting immunity against viral antigens alone, neglecting the contribution of mosquito-derived factors to infection. During blood feeding, infected female mosquitoes continuously secrete saliva containing both virions and salivary proteins with vasodilatory, anticoagulant, and immunomodulatory properties. These effects facilitate feeding but can also increase vascular permeability, promote the influx of virus-susceptible immune cells to the bite site, and enhance viral replication in resident skin cells, exacerbating dissemination and disease severity [13]. In recent years, targeting mosquito saliva components has been explored as a potential vaccine strategy [14,15,16]. By targeting salivary proteins, these vaccines aim to prime adaptive immune responses that are recalled when salivary antigens are deposited during mosquito feeding or experimental challenges. This may alter the local immune environment at the inoculation site, potentially favouring a more antiviral response and reducing early virus establishment and downstream systemic spread. This approach could also have the added benefit of providing protection against multiple viruses transmitted by the same vector.

PepTcell Ltd., the parent company of ConserV Bioscience Ltd., previously demonstrated safety and immunogenicity of another mosquito salivary subunit vaccine, AGS-v PLUS, which targets conserved mosquito salivary proteins [17,18]. AGS-v PLUS also includes peptides derived from salivary proteins conserved in *Aedes* mosquitoes, and vaccination has been shown to modify the human skin’s transcriptional response to controlled *Aedes* mosquito biting [19]. Building on this approach, in this study, a peptide-based vaccine targeting the saliva of *Aedes* and *Culex* mosquitoes (SAL) was developed. Regions from salivary proteins that were conserved in either *Aedes* and/or *Culex* were selected and analysed using a proprietary algorithm to select those with predicted broad HLA coverage for MHC class I presentation. The selection was narrowed down based on in vivo cellular and humoral immune responses, and the final selection of peptides was evaluated for efficacy, alone or co-administered with viral VLPs, against two arboviral targets, CHIKV and ZIKV. The combination of salivary and viral antigens aimed to generate a dual-pronged strategy, combining virus-specific immunity with an adaptive response to vector-derived salivary antigens that may favour antiviral immune conditioning at the inoculation site.

## 2. Materials and Methods

### 2.1. Identification of T-Cell-Reactive Sequences

A literature search and mining of the NCBI database were conducted to identify known proteins found in the saliva of *Aedes* and *Culex* female mosquitoes reported as “secreted” or “putative secreted”. CLUSTAL omega [20] was used to perform multiple sequence alignments which were conducted separately for each salivary protein. The resulting consensus sequence for each protein was analysed for the presence of reactive T-cell epitopes for multiple human HLA-I alleles and mouse MHC-I using a proprietary algorithm (PepTcell Ltd. (London, UK), parent company of ConserV Bioscience Ltd.). The algorithm analyses the structural affinity of a peptide for a particular HLA/MHC allele and uses data extracted from a set of reactive vs non-reactive epitopes identified empirically to determine if a sequence of amino acids contains a T-cell “reactive” or “self” epitope for that HLA. Protein regions (peptides) of <40 amino acid long, with at least 70% conservation for each consecutive residue, and with clusters of predicted T-cell epitopes for multiple HLAs were selected and further analysed using NCBI BLAST [21] to identify homology to known human and murine proteins. Peptides with at least seven consecutive amino acids shared with humans/mice were discarded to reduce the risk of developing an autoimmune response post-vaccination. A set of forty peptides was selected and manufactured as acetate peptides (Genscript, Oxford, UK).

### 2.2. Viruses

The BNI-CHIKV_899 strain (GenBank accession no. FJ959103.1) was generously provided by Prof. Christian Drosten (University of Bonn, Germany). Virus stocks were propagated in *Aedes* albopictus mosquito-derived C6/36 cells, a kind gift from Prof. Alain Kohl (Liverpool School of Tropical Medicine, UK), as previously described [16]. PRVABC-59 Zika virus (ZIKV) was kindly provided by Kris Subramaniam at the University of Liverpool (UK). ZIKV was propagated in Vero cells (ATCC CCL-81, LGC Standards, Teddington, UK) for 4 days at 37 °C in a humidified 5% CO_2_ incubator in complete Dulbecco’s Modified Eagle Medium (DMEM) (Gibco, Thermo Fisher Scientific, Loughborough, UK) supplemented with 10% Foetal Bovine Serum (FBS) (Sigma-Aldrich, Gillingham, UK), 10% Tryptose Phosphate Broth (TPB) (Gibco, Thermo Fisher Scientific, Loughborough), 5 mL Pen/Strep (10,000 U/mL, Gibco, Thermo Fisher Scientific, Loughborough), and 5 mL GlutaMAX (Gibco, Thermo Fisher Scientific, Loughborough). CHIKV and ZIKV viral titres were quantified on Vero cells by either the TCID50 assay or plaque assay, as shown below.

### 2.3. Mice

For immunogenicity studies, all animal work was performed at the University of Leicester (UK) at the Preclinical Research Facility managed by the Division of Biomedical Services that operates under the UK Home Office license to AMC (P6DCE1A76, 6 August 2018) and under an establishment licence to the University of Leicester (X1798C4D2). First, 7–9-week-old female C57BL/6J mice were either reared in-house or purchased from Charles River Laboratories UK (Margate, UK) and acclimatised as per requirements.

For the ZIKV efficacy studies, immunocompromised *Ifnar1-/-* mice on a C57BL/6 background were used, as they are susceptible to ZIKV and can develop clinical signs of infection [22]. Mice were reared in-house at the University of York (UK), where studies were conducted following ethical approval by the University of York Animal Welfare and Ethical Review Body (AWERB) and under UK Home Office licence PP0258562.

CHIKV efficacy studies were performed in C57BL6 mice in the biosafety level 3 (BSL-3) facilities of the Rega Institute for Medical Research (KU Leuven, Belgium), in accordance with institutional regulations, under licenses AMV 30112018 SBB 219 2018 0892 and AMV 23102017 SBB 219 2017 0589. Procedures were conducted with the approval of the Ethical Committee of the University of Leuven (license DMIT-026/2025), following the Federation of European Laboratory Animal Science Associations (FELASA) guidelines.

### 2.4. Collection of Mosquito Saliva

*Ae. aegypti* Liverpool strain mosquitoes [16] were housed in an insectary at 28 °C and 75% Relative Humidity (RH) with a 14/10 day/night light cycle. Larvae were reared in purified water and fed on ground TetraMin flake fish food (Tetra Werke, Melle, Germany). Adults were fed 10% sucrose (Sigma-Aldrich, Gillingham, UK) *ad libitum*, and blood-fed on defibrinated horse blood (TCS Biosciences, Buckingham, UK) using a Hemotek AS6W1-3 (Hemotek, Blackburn, UK) membrane feeder covered with Parafilm M Sealing Film (Bemis/Heathrow Scientific, Neenah, WI, USA). Mosquitoes used for salivation were 4 to 8 days post-emergence. Emerging adult mosquitoes were maintained on a 10% (w/v) sucrose solution *ad libitum.* For the salivation of mosquitoes, the mosquito proboscis was placed in a p10 tip containing 0.5 μL immersion oil (Cargille Laboratories, Cedar Grove, NJ, USA). Mosquitoes were then left to salivate for up to an hour before tips were placed in an Eppendorf tube and centrifuged. Saliva droplets were then pooled and stored at −70 °C. Before use, droplets of saliva were carefully pipetted out of the oil under the microscope and diluted in PBSA (PBS with 0.75% Bovine Serum Albumin (BSA), Fraction V (Roche, Welwyn Garden City, UK)). For each challenge experiment, *Ae. aegypti* saliva was collected by salivation/probing and pooled before dilution into the virus inoculum. Saliva input was standardised by mosquito equivalent rather than by measured total protein concentration. Each mouse received the equivalent of saliva from two mosquito probing/salivation events. Within each challenge experiment, the same pooled saliva preparation was distributed equivalently across all vaccine groups, so that the saliva pool and mosquito-equivalent dose were not confounded with the vaccination group.

### 2.5. Immunisations and Viral Challenges

#### 2.5.1. Salivary Peptide Immunogenicity Screening

For the salivary peptide immunogenicity screening studies, 40 salivary peptides were manufactured by Genscript (Oxford, UK) and reconstituted in dimethyl sulfoxide or water (Sigma-Aldrich, Gillingham, UK), as per the manufacturer’s recommendations, to reach a 10 mM stock solution of each peptide. Peptides were split into 8 pools containing 5 peptides per pool and were diluted in water to 0.2 mM of each peptide in the mix. Immediately prior to administration, the peptide solution was emulsified in the adjuvant Montanide ISA-51 (Seppic, Castres, France), composed of a white mineral oil and mannide monooleate as the non-ionic surfactant. Each animal received a 0.1 ml subcutaneous dose of the water-in-oil emulsion containing 10 nmol of each peptide as a prime and boost injection two weeks apart. Each group included n = 4 female C57BL/6 mice 7–9 weeks old (Supplementary Table S1 Part 1).

#### 2.5.2. Immunogenicity of the Final SAL Formulation

To assess the immunogenicity of the final SAL peptide mix, n = 6 (3 females, 3 males) C57BL/6 mice were immunised using the same vaccination regime. In all studies, mice were humanely euthanised two weeks after the boost and spleens and serum were collected for immunological analysis (Supplementary Table S1 Part 1).

#### 2.5.3. Vaccination Regimen for Efficacy Studies

For efficacy studies, mice were immunised subcutaneously twice, two weeks apart, with 0.1 ml adjuvanted doses of placebo (water), 0.5 ug of CHIKV VLPs or ZIKV VLPs (The Native Antigen Company, Kidlington, UK), the mix of the salivary peptides (SAL) (10 nmol of each peptide), or VLP + SAL. A single VLP dose of 0.5 µg was used throughout, administered identically to all VLP-containing groups. This dose was deliberately selected to be partially rather than fully protective, so that any additive contribution of the SAL component would be detectable. A formal VLP dose-ranging study was not performed. Animals receiving both VLPs and SAL received separate injections of VLPs and SAL on opposite flanks. Two weeks after the boost, mice were anaesthetised with isoflurane (Iso-Vet Piramal Critical Care, Morpeth, UK) and challenged with ZIKV or CHIKV, as described below. All efficacy studies were performed blinded. The ZIKV and CHIKV challenge teams received vaccine formulations under coded group labels, and vaccine group identities were not disclosed until after data analyses were complete (Supplementary Table S1 Part 1).

#### 2.5.4. CHIKV Challenge and Sampling

Female mice were used for the CHIKV challenge studies to maintain consistency within the established model, reduce biological variability, and limit animal use in accordance with the 3R principles. Mice were injected subcutaneously into the left hind footpad with an inoculum of 20 μL containing 10^6^ plaque-forming units (PFUs) of CHIKV diluted in medium with 2% FBS (Thermo Fisher Scientific, Brussels, Belgium) and 2 μL of mosquito saliva, using 25-gauge needles (Henke-Ject, Tuttlingen, Germany). Footpad swelling was assessed daily by measuring the width and height of both the infected and contralateral, uninfected footpad, using a digital calliper (Manutan, Verwood, UK). Body weight was monitored daily after virus infection. Mice were euthanised at 2 or 7 days post-infection (dpi) (n = 8 mice per group, per timepoint) by intraperitoneal injection with Dolethal (Vétoquinol, Towcester, UK). Blood was collected via cardiac puncture, and serum was obtained after centrifugation at 9400× *g*, +4 °C, for 10 min. Tissues, including the ipsilateral and contralateral ankle joint, were harvested for quantification of viral RNA and infectious virus titres (Supplementary Table S1 Part 2). Joint tissue was homogenised in Precellys tubes with 2.8 mm zirconium oxide beads (Bertin Instruments/Bertin Technologies, Montigny-le-Bretonneux, France) and medium with 2% FBS. An automated homogenizer (Precellys24, Bertin Instruments/Bertin Technologies, Montigny-le-Bretonneux, France) was used for homogenization in two cycles at 5430× *g* for 20 s, with a 20 s interval, after which homogenates were centrifuged (21,100× *g*, 10 min, +4 °C).

#### 2.5.5. ZIKV Fixed-Timepoint Challenge and Sampling

In the ZIKV model, male mice were injected into the hind footpad with 750 PFU ZIKV and 2 μL mosquito saliva in a total volume of 20 μL in PBSA (PBS (Invitrogen, Thermo Fisher Scientific, Loughborough, UK) with 0.75% w/v BSA) using U-100 Micro-Fine insulin needles (BD, Plymouth, UK). Following injection, mice were returned to cages and monitored until recovery. At 2 dpi, n = 8 mice per group were euthanized and spleens, popliteal lymph nodes, and skin at the inoculation site were collected in RNAlater (Thermo Fisher scientific, Altrincham, UK) for RNA extraction, along with blood serum samples. At 4 dpi, n = 8 mice per group were euthanised and the skin at the inoculation site, spleen, and half brain were collected in RNAlater, along with blood serum samples (Supplementary Table S1 Part 2).

#### 2.5.6. ZIKV Clinical Sign and Survival Study

For the clinical sign survival experiment, female mice were inoculated subcutaneously in the dorsal left foot skin with 750 PFU ZIKV (2 μL) mixed with 2 μL mosquito saliva (total 4 μL) using custom-made point 4 style 33-gauge microneedles (Hamilton^®^, Bonaduz, Switzerland) and a 5 μL glass 75 RN Hamilton syringe. Mice were monitored three times daily for up to 15 days for clinical signs of ZIKV infection [23]. Animals showing ≥15% body weight loss or predefined clinical signs of ZIKV disease were humanely culled (Supplementary Table S1 Part 2). Sex allocation reflected animal availability from the *Ifnar1*-deficient breeding colony and was guided by the 3R considerations to avoid unnecessary breeding or culling of otherwise suitable animals. Fixed-timepoint and clinical-monitoring experiments were therefore performed as independent, internally sex-matched cohorts, and no analyses involved direct comparison between male- and female-derived datasets. The inoculation method also differed for technical reasons: male mice in the 2 and 4 dpi cohorts had dorsal foot skin that was too tough for reliable delivery using 33-gauge Hamilton microneedles. These animals were therefore inoculated into the footpad using U-100 Micro-Fine insulin needles (Becton, Dickinson and Company, Wokingham, UK), while maintaining a small inoculation volume.

#### 2.5.7. Mosquito Saliva Standardisation Across Challenge Experiments

Across efficacy studies, saliva input was standardised, as described in Section 2.4. Briefly, each challenged mouse received the equivalent of saliva from two *Ae. aegypti* probing/salivation events, consistent with the mosquito saliva challenge model used previously [16]. Within each challenge experiment, pooled saliva was distributed equivalently across vaccine groups, preventing the saliva pool or mosquito-equivalent dose from being confounded with the vaccination group (Supplementary Table S1 Part 2). Total salivary protein concentration was not measured.

### 2.6. Viral Titration by Endpoint Dilution Assay

Infectious virions were determined on African green monkey kidney epithelial cells (Vero cells, ATCC CCL-81, LGC Standards, Teddington, UK). KU Leuven cultured the cells in MEM + 10% FBS, 1% L-Glutamine, 1% NEAA, and 1% sodium bicarbonate (Merck Life Science, Overijse, Belgium), whereas at the University of York cells were cultured in DMEM (Sigma-Aldrich, Gillingham, UK) supplemented with 10% FBS (Sigma-Aldrich, Gillingham, UK) at 37 °C with 5% CO_2_. Cells were pre-seeded in 96-well plates at a density of 2.5 × 10^4^ cells/well. After overnight incubation (37 °C, 5% CO_2_), cell monolayers were washed once with PBS (200 μL/well) (Sigma-Aldrich, Gillingham, UK). At the University of York, this was followed by the addition of 50 μL of ten-fold serial dilutions (10^−1^ to 10^−7^) of tissue homogenates or cell culture supernatants to the cell culture in triplicate. Plates were incubated at 37 °C for 1 h, after which 150 μL of complete medium was added. Cells were monitored microscopically daily for virus-induced cytopathogenic effects (CPEs), with uninfected cells serving as negative controls. Following a 4-day incubation period at 37 °C, supernatants were discarded, and cells were fixed with 10% paraformaldehyde (50 μL/well, 30 min, room temperature) (Sigma-Aldrich, Gillingham, UK), stained with 1% crystal violet (50 μL/well, 30 min, room temperature) (Sigma-Aldrich, Gillingham, UK), rinsed with tap water, and dried. A similar protocol was followed at KU Leuven with the exception that the 10-fold dilutions were performed directly on the plate (20 μL sample mixed with 180 μL of media) and the incubation was 3 days. The TCID50/mL (tissue culture infectious dose 50%), representing the viral concentration required to infect 50% of the cells, was determined using the Reed and Muench method [24]. The limit of quantification (LOQ) was defined as the lowest viral titre that could be reliably quantified using this method, considering tissue weights and buffer volumes.

### 2.7. Splenocyte Cytokine FluoroSpot

Splenocytes were isolated by mashing the spleens through a 70 μm cell strainer. Red blood cells were lysed (red blood cell lysis buffer, Sigma-Aldrich, Gillingham, UK) followed by washing the splenocyte cell suspension three times with RPMI-1640 (Gibco, Thermo Fisher Scientific, Loughborough, UK) before counting using a haemocytometer or Countess II (Thermo Fisher Scientific, Loughborough, UK). Then, 4 × 10^5^ cells were added per well to pre-coated IFN-γ, IL-2, and TNF-α FluoroSpot 96-well plates (Mabtech, Nacka Strand, Sweden) and incubated with 2 µM of each peptide, media only (negative control), or 5 µg/mL Concanavalin A (Sigma-Aldrich, Gillingham, UK) as a positive control. Anti-CD28 antibody (Mabtech, Nacka Strand, Sweden) was added at a 1:1000 dilution to provide the necessary co-stimulatory molecules to support T-cell activation. Plates were incubated for 40–46 h at 37 °C and 5% CO_2_ before proceeding to the detection of the analytes using fluorescently labelled antibodies and following the manufacturer’s instructions (Mabtech, Nacka Strand, Sweden). Plates were left to air dry and spot-forming units (SFUs) were counted using the IRIS2 FluoroSpot plate reader (Mabtech, Nacka Strand, Sweden). SFUs for each stimulation were normalised by subtracting the SFU counts in the media-only wells (negative control). The counts were adjusted to present the number of SFU/million cells. The median of the group was calculated for statistical comparison.

### 2.8. Total Immunoglobulin ELISA

Peptide-specific antibodies in sera were measured using an indirect ELISA, as peptide-specific capture antibodies were not available to allow for a sandwich format. Peptide antigens were therefore immobilised directly onto the plate and bound mouse serum immunoglobulin was detected using an HRP-conjugated anti-mouse Ig secondary antibody. Plates were coated overnight at +4 °C with 100 μL of individual peptides/well at 2 µM in PBS (Gibco, Thermo Fisher Scientific, Loughborough, UK). The following day, plates were washed twice with 0.05% Tween-PBS (PBS-T) (Tween-20 and PBS, Sigma-Aldrich, Gillingham, UK), before blocking for 1 h with 200 μL of 1% BSA in PBS (BSA and PBS from Sigma-Aldrich, Gillingham, UK). Plates were washed four times with PBS-T, followed by the addition of 100 μL of serum samples diluted 1:200 in 1% BSA-PBS. This dilution was selected to retain a measurable peptide-specific antibody signal while reducing non-specific background from serum components. After two hours of incubation, plates were washed six times with PBS-T and 100µ of goat anti-mouse Ig HRP-conjugated antibody (Sigma-Aldrich, Gillingham, UK) was added (1:5000 in 1% BSA-PBS) and incubated for 1 h. Plates were washed eight times before adding 100 μL of TMB solution (Biolegend, London, UK) and allowing the reaction to develop for 30 min. Development was stopped by the addition of 100 μL of 1N H_2_SO_4_ (Fisher, Loughborough, UK). A plate reader (Thermo Multiskan EX, Thermo Electron Corporation/Fisher Scientific, Loughborough, UK) was used to measure absorbance at 450 nm and 570 nm. For each well, absorbance at 570 nm was subtracted from absorbance at 450 nm to correct for optical background arising from interfering substances or plate imperfections. Serum samples were analysed in duplicate. The mean corrected absorbance of the duplicate wells was calculated to generate a single value for each mouse and peptide. Group medians and interquartile ranges were calculated from these biological replicate values and used for graphical presentation and statistical analysis.

### 2.9. RNA Extraction, Purification and Quantification

Viral RNA was isolated from homogenized joint tissue or from serum of CHIKV-infected mice using the NucleoSpin RNA virus kit (Macherey-Nagel, Hœrdt, France) following the manufacturer’s protocol. Viral RNA was quantified by qRT-PCR in a total reaction volume of 25 μL, composed of 13.94 μL of nuclease-free water (Promega, Southampton, UK), 6.25 μL of master mix (Eurogentec, Seraing, Belgium), 0.375 μL of each primer (Table 1) at a final concentration of 150 nM, 0.0625 μL of reverse transcriptase (Eurogentec, Seraing, Belgium) and 3 μL of RNA template. Reactions were run on a QuantStudio 5 Real-Time PCR system (ThermoFisher Scientific, Loughborough, UK) with the following thermal cycling conditions: 30 min at 48 °C, 10 min at 95 °C, 40 cycles of 15 s at 95 °C and 1 min at 60 °C. A standard curve was derived using a ten-fold serial dilution of an in-house-generated CHIKV Nsp1-containing plasmid based on the pJET vector (ThermoFisher Scientific, Brussels, Belgium). The limit of quantification (LOQ) was defined as the lowest amount of viral RNA that could be quantitatively determined with precision and accuracy in at least 95% of the qRT-PCR assays, considering the tissue weight and buffer volumes.

Serum, skin, half brain, spleen, and popliteal lymph node samples were collected from ZIKV-infected mice, immersed in RNAlater, and stored at −70 °C. Samples were homogenised in TRIzol (Invitrogen, ThermoFisher Scientific, Loughborough, UK) and shaken with stainless-steel beads using a TissueLyser (QIAGEN, Manchester, UK) at 50 Hz for 10 min, followed by phase separation with chloroform (Thermo Fisher Scientific, Loughborough, UK). RNA from the aqueous phase was purified using Purelink RNA Mini Kit columns (Thermo Fisher Scientific, Loughborough, UK), as per the manufacturer’s protocol. Using the RNase-free DNase set (QIAGEN, Manchester, UK), an on-column DNA step was incorporated between the initial and subsequent washes. This step aims to degrade any genomic DNA contamination. Each spin column received 80 μL of DNase mixture, consisting of 10 μL of DNase and 70 μL of reaction buffer, and was incubated at room temperature for 15 min. Following the washes, the column underwent a 1 min centrifugation to ensure it was dry before RNA elution. DNase treatment was performed only on lymph node samples. RNA was converted to cDNA using the High-Capacity RNA-to-cDNA Kit (Applied Biosystems, ThermoFisher Scientific, Loughborough, UK). Reactions were prepared using up to 2 μg of total RNA, as per the manufacturer’s instructions. The resulting cDNA was diluted 1:5 with nuclease-free water and stored at −70 °C for further analysis. Quantitative (q) PCR was undertaken using PerfeCTa SYBR^®^ Green FastMix (Quantabio, Beverly, MA, USA). Each biological replicate was run in at least three technical replicates. A standard curve was generated using a 10-fold serial dilution of a PCR-generated standard, as previously described [25]. Reactions were performed on an QuantStudio 7 Pro system (Thermo Fisher Scientific, Loughborough, UK) with the following cycling conditions: 95 °C for 3 min, followed by 25–40 cycles of 95 °C for 3 s and 60 °C for 30 s, ending with a melt curve analysis to verify primer specificity. The cycle threshold (Ct) values were automatically determined by the QuantStudio software (ThermoFisher connect platform), and relative gene expression was normalised to the 18S housekeeping gene. Primers were designed using Primer3 (version 4.1, access date 8 January 2025 https://primer3.ut.ee), are shown in Table 1, and were manufactured by Integrated DNA Technologies (Leuven, Belgium). Analysis of RT-qPCR data was done with Microsoft Excel using the median of the technical replicates and normalising them to the median of the technical replicates of the housekeeping gene 18S, which is not altered by ZIKV infection.

### 2.10. Statistical Analysis

All statistical analyses were performed using Prism v.10.6.1 (Graphpad, San Diego, CA, USA). The cutoff value used to determine significance was *p* ≤ 0.05 for all tests. Normality was assessed using a D’Agostino and Pearson test. For comparisons between two groups, a non-parametric Mann–Whitney U test was used. Ordinary One-Way ANOVA with Tukey’s correction was performed for comparisons between more than two groups of normally distributed data. Non-parametric Kruskal–Wallis tests with Dunn’s correction were used for comparisons between more than two groups of non-normally distributed data. An analysis of survival curves was conducted using the log-rank (Mantel–Cox) test. Individual tests performed are noted in each figure legend.

## 3. Results

### 3.1. Screening of Mosquito Salivary Peptides

A total of forty peptides originating from proteins in the saliva of *Aedes* and/or *Culex* mosquitoes were selected in silico based on predicted immunogenicity and conservation. Mice were immunised with pools of five peptides to screen for immunogenicity in vivo. ELISA was used to detect peptide-specific antibody responses in serum, whereas the FluoroSpot assay was used to quantify cytokine release by splenocytes upon re-stimulation with the peptide antigens. All data were compiled (Supplementary Table S2), and nine peptides were selected based on the strength and consistency of the induced immune response (inducing antibodies and/or secretion of ≥2 cytokines) and the protein each peptide originated from to ensure a broad portfolio of putative secreted targets within the saliva of *Culex* and *Aedes* female mosquitoes (Table 2).

**Figure 1:**
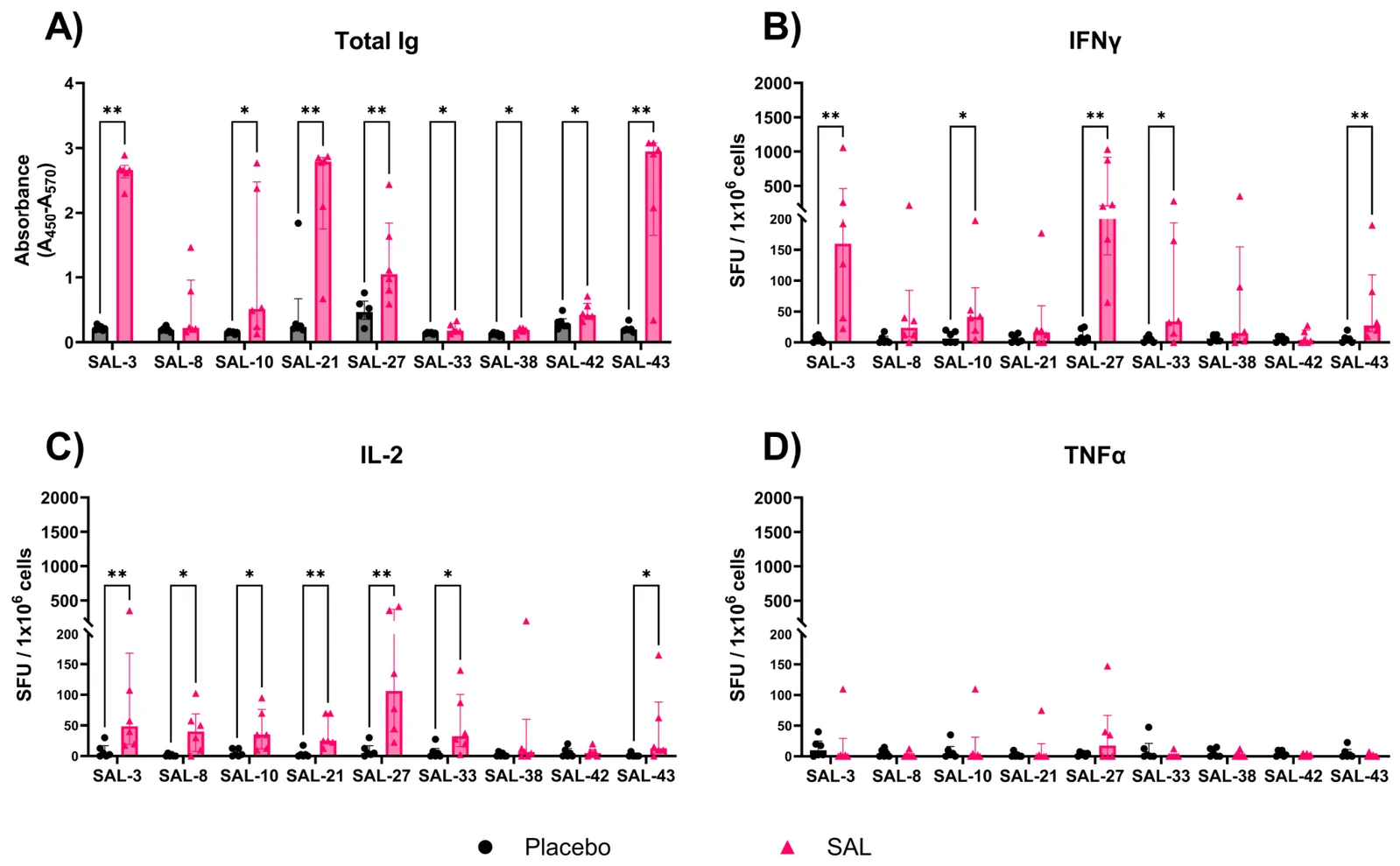
Immune responses to SAL vaccine. C57BL/6 mice were subcutaneously injected at days 0 and 14 with saline (placebo) or a 10 nmol equimolar mix of SAL peptides in Montanide ISA-51 adjuvant, then culled at day 28 (n = 3 males + n = 3 females/group). (**A**) Total serum immunoglobulin (Ig) against each SAL peptide was determined by ELISA, presented as absorbance normalised to background (A_450nm_–A_570nm_). (**B**–**D**) IFNγ, IL-2 and TNFα cytokine responses were assessed by FluoroSpot in splenocytes re-stimulated with the SAL peptides, presented as spot-forming units (SFU) per million cells. Triangles and dots represent individual mice and bars (black = placebo group, pink = SAL-vaccinated group) represent median with interquartile range. A Mann–Whitney U test was performed to compare the medians of the two groups for each peptide. * = *p* ≤ 0.05, ** = *p* ≤ 0.01.

The mix of the selected nine peptides was termed “SAL”. Peptides were formulated in an equimolar ratio and mixed with Montanide ISA-51 adjuvant to evaluate their immunogenicity in vivo and ensure that all peptides continued to induce immune responses without interference or immunodominance (Figure 1). Total Ig ELISA results (Figure 1A) indicated that, with the exception of the SAL-8 peptide, all other peptides induced significantly higher antibody responses compared to placebo, with SAL-3, SAL-21 and SAL-43 inducing the highest titres, and SAL-27 to a lesser extent.

Splenocytes from SAL-vaccinated mice showed a significantly increased number of IFNγ SFUs in response to SAL-3, -10, -27, -33 and -43 compared to splenocytes from mice vaccinated with the placebo (Figure 1B). Likewise, significantly higher IL-2 SFUs were recorded by splenocytes of mice vaccinated with SAL in response to all peptides except SAL-38 and SAL-42 (Figure 1C). No TNFα SFUs were detected after in vitro exposure of splenocytes with any of the peptides (Figure 1D). Some peptide-specific differences were noted between the responses induced to the final selection of nine peptides and those induced when administered as pools of five peptides (Supplementary Table S2). The antibody responses were higher for SAL-21 when administered as the SAL final mix; however, lower cellular responses were observed for peptides SAL-33, SAL-42 and SAL-43 when administered as the SAL final mix compared to when administered as the pool of five peptides during screening. Nevertheless, peptides SAL-3, -21 and -43 continued to induce very robust antibody responses (Figure 1A) and SAL-3 and -27 peptides induced the strongest cellular stimulation (Figure 1B).

### 3.2. Efficacy of SAL in Combination with VLPs in a CHIKV Infection Model

**Figure 2:**
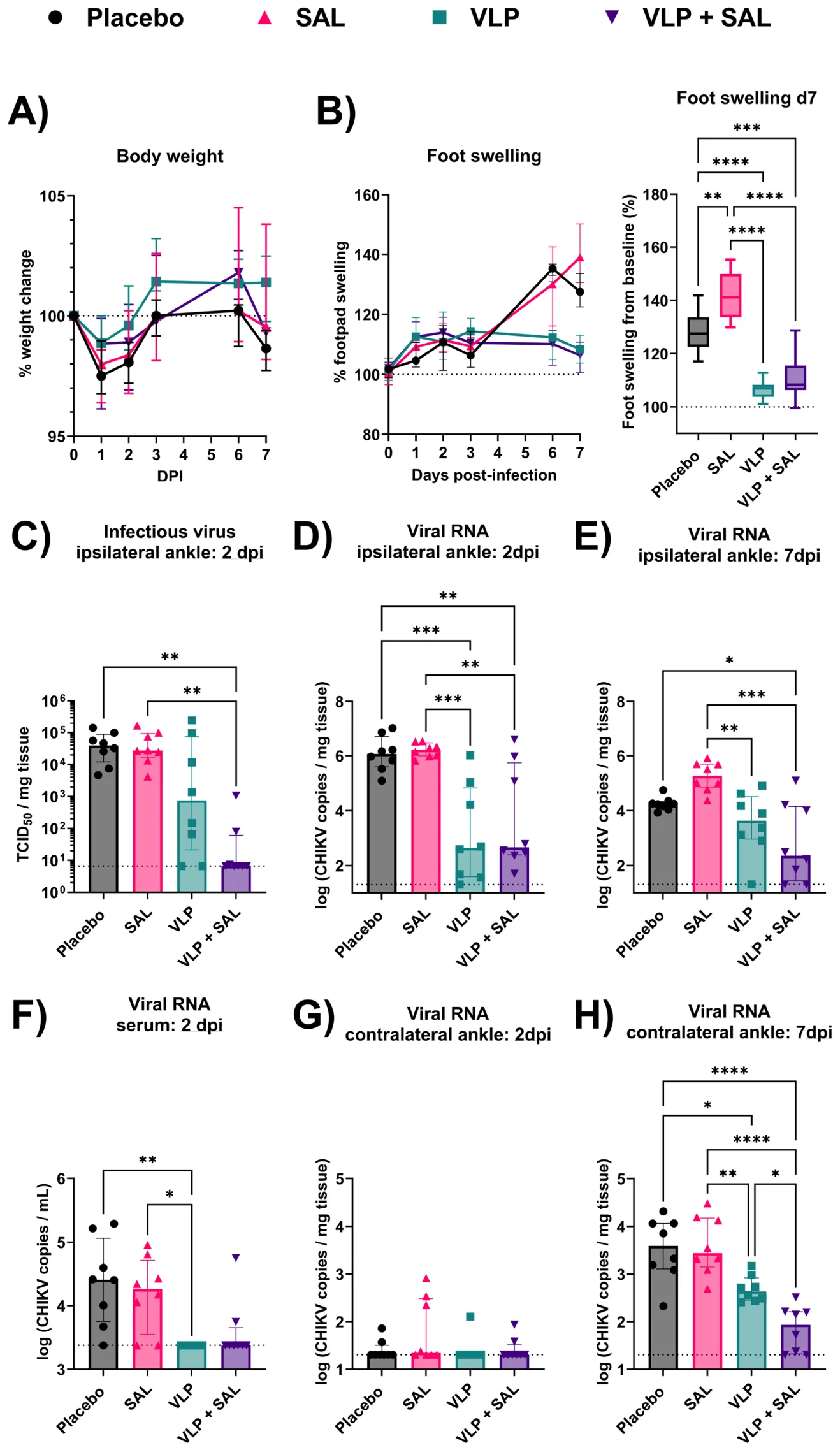
**Efficacy of SAL, VLP and combined SAL + VLP vaccines against CHIKV infection.** Female C57BL/6 mice were subcutaneously injected on days 0 and 14 with 100 μL of adjuvanted saline (placebo, black/grey), adjuvanted SAL vaccine (10 nmol of each peptide, pink), adjuvanted CHIKV VLPs (0.5 ug, green), or both adjuvanted SAL and VLPs in opposite flanks (purple). Mice were then challenged subcutaneously in the footpad with 1 × 10^6^ PFU CHIKV 899 and mosquito saliva on day 28, across three independent studies. Half of the mice were euthanised 2 days post-infection (dpi) and the other half were euthanised 7 dpi (total n = 16 per active treatment group and n = 8 for placebo, per timepoint). (**A**) Body weight post-infection was recorded, presented as percentage change from baseline (day of challenge). Line graph represents group median with interquartile range (IQR). (**B**) Foot swelling was measured by callipers and presented as percentage change from baseline over time or as percentage change on day 7 alone (One-Way ANOVA with Tukey’s correction). Line graph represents group median + IQR. Box plots represent median with 9–95 percentiles. (**C**) Infectious virus in the ipsilateral ankle at 2 dpi was determined by viral titration in Vero cells, presented as TCID50 per mg of tissue collected (Kruskal–Wallis with Dunn’s correction). Viral RNA was quantified by qPCR, presented as copies of CHIKV RNA per mg of tissue collected from (**D**) ipsilateral ankle at 2 dpi (One-Way ANOVA with Tukey’s correction), (**E**) ipsilateral ankle at 7 dpi (One-Way ANOVA with Tukey’s correction), (**F**) serum at 2 dpi (Kruskal–Wallis with Dunn’s correction), (**G**) contralateral ankle at 2 dpi (Kruskal–Wallis with Dunn’s correction), or (**H**) contralateral ankle at 7 dpi (One-Way ANOVA with Tukey’s correction). Points represent individual mice and bars represent median with interquartile range. Dotted lines represent the assay limits of detection. * = *p* ≤ 0.05, ** = *p* ≤ 0.01, *** = *p* ≤ 0.005 and **** = *p* ≤ 0.001.

The efficacy of SAL peptides delivered alone or in combination with CHIKV VLPs was assessed in a CHIKV disease mouse model. Mice were immunised subcutaneously with adjuvanted placebo, SAL, CHIKV VLPs, or CHIKV VLPs + SAL (administered on opposite flanks). Following CHIKV infection, body weight remained relatively stable across all groups (Figure 2A). Descriptively, median peak weight loss was 1.4% in both CHIKV VLP-containing groups, compared with 2.7% in the placebo group and 2.3% in the SAL group.

Daily monitoring of foot swelling, the main clinical sign of this disease model, showed that peak foot swelling occurred at 6–7 dpi in the placebo group (Figure 2B). At 7 dpi, there was a pronounced and significant inhibition of swelling in the groups immunised with CHIKV VLPs (mean increase of 7% from baseline) or CHIKV VLP + SAL (11%) compared to the placebo (28%). In contrast, SAL alone did not significantly affect peak foot swelling compared to the placebo.

To evaluate the impact of vaccination on viral replication, viral load in the infected (ipsilateral) ankle joint was determined by quantification of viral RNA at 2 dpi by qPCR (Figure 2D). CHIKV VLP and CHIKV VLP + SAL vaccinations significantly reduced the levels of viral RNA compared to the placebo, while SAL vaccination alone did not. However, when quantifying infectious viral load by endpoint titrations, only vaccination with CHIKV VLP + SAL resulted in a significant reduction in infectious virions at the infected joint compared to the placebo (Figure 2C). Infectious virus could not be quantified in over a third of CHIKV VLP + SAL vaccinated mice, due to being below the limit of quantification of the assay. This would suggest a role for SAL in reducing infectious viral load at the site of the inoculation when combined with CHIKV VLPs. By 7 dpi (Figure 2E), only mice immunised with CHIKV VLPs + SAL had significantly lower viral RNA in the ipsilateral ankle compared to placebo, confirming that the reduction in viral load observed on day 2 resulted in lower viral replication and, consequently, lower viral RNA was detected five days later.

Viral load in serum and in the contralateral ankle joint (the joint opposite to the joint that received the viral inoculum) was measured to determine the effect of vaccination on systemic viral dissemination. At 2 dpi, serum viral RNA was significantly lower in CHIKV VLP-vaccinated mice than in placebo-treated mice. Viral RNA values in the CHIKV VLP + SAL group were also very low, with the group median close to the assay limit of detection (Figure 2F). In the contralateral ankle joint (Figure 2G,H), viral RNA was largely undetectable at 2 dpi in all groups but became evident by 7 dpi as infection progressed and the virus was disseminated. At this timepoint, mice immunised with CHIKV VLPs or CHIKV VLP + SAL had less viral RNA in the contralateral ankle compared to the placebo group. Moreover, the mean number of viral RNA copies/mg of tissue was also significantly lower in the CHIKV VLP + SAL-vaccinated group compared to CHIKV-VLP-alone group (112 and 588 copies, respectively, *p* = 0.0185) highlighting the protective additive effect of SAL when administered with VLPs.

Collectively, these results indicate that combining SAL with CHIKV VLPs provides the same local benefits as VLPs alone, while also significantly improving systemic efficacy by reducing viral dissemination.

### 3.3. Efficacy of SAL in Combination with VLPs in a ZIKV Infection Model

The efficacy of SAL, either alone or in combination with ZIKV VLPs, was also assessed in a ZIKV challenge model. Mice were either culled 2 or 4 dpi for tissue collection and virological analysis or monitored for 15 days to assess clinical outcomes.

**Figure 3:**
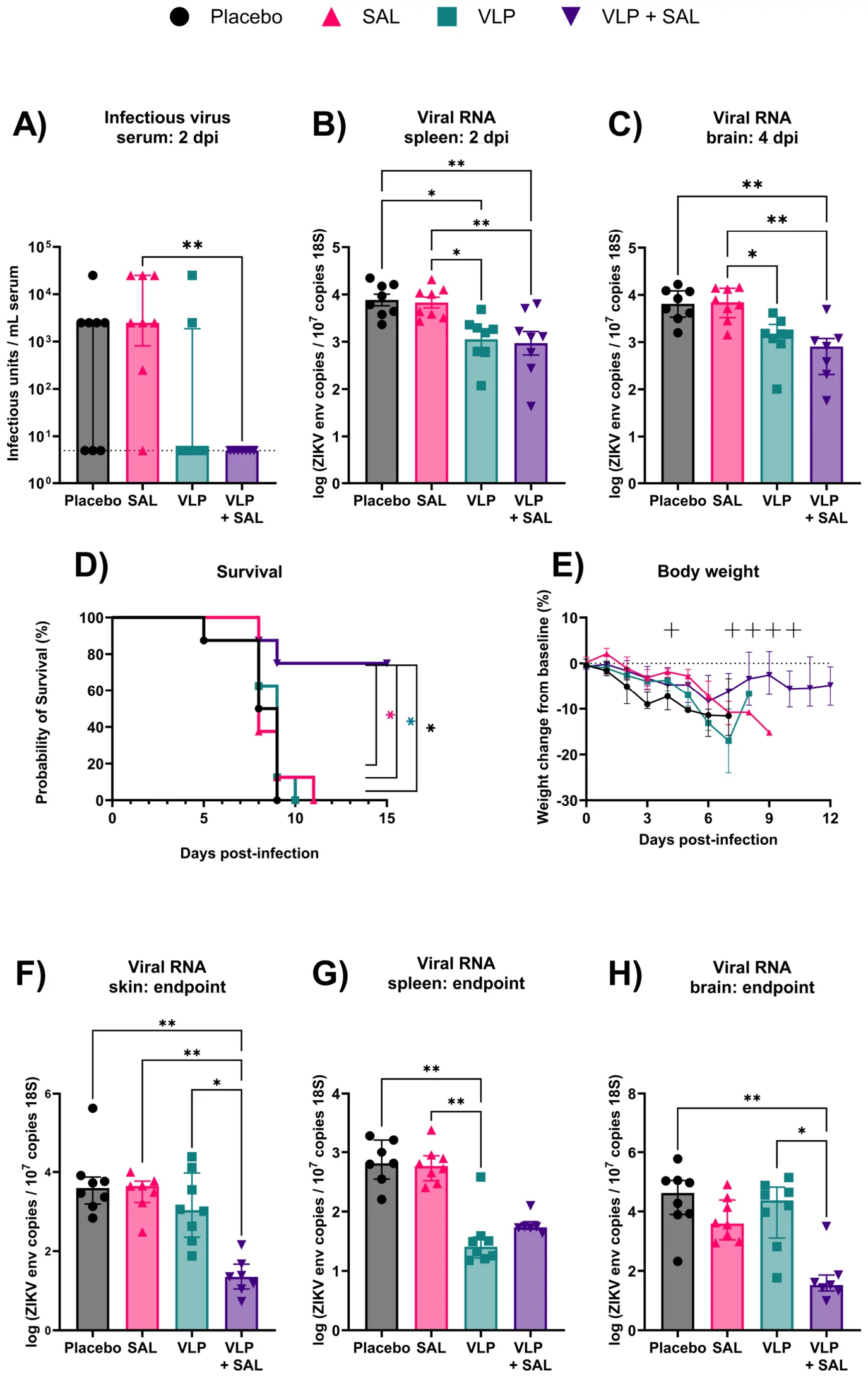
Efficacy of SAL, VLP and combined SAL + VLP vaccines against ZIKV infection. Ifnar1-/- mice were subcutaneously injected on days 0 and 14 with 100 μL of adjuvanted saline (placebo, grey bars or black lines), adjuvanted SAL vaccine (10 nmol of each peptide, pink bars or lines), adjuvanted ZIKV VLPs (0.5 ug, green bars or lines), or both adjuvanted SAL and VLPs in opposite flanks (purple bars or lines). For fixed-timepoint studies, male mice were challenged subcutaneously in the footpad with 750 PFU ZIKV and mosquito saliva on day 28. Half the mice were euthanised 2 days post-infection (dpi) and the other half were euthanised 4 dpi (total n = 8 per group, per timepoint). (**A**) Infectious virus in serum at 2 dpi was determined by endpoint dilution assay in Vero cells, presented as infectious virus per mL serum collected (Kruskal–Wallis with Dunn’s correction). Viral RNA was quantified by qPCR in (**B**) the spleen at 2 dpi (One-Way ANOVA with Tukey’s correction) and (**C**) the brain at 4 dpi (Kruskal–Wallis with Dunn’s correction), presented as copies of ZIKV envelope (env) RNA per 10^7^ copies of housekeeping gene 18S. Points represent individual mice and bars represent median with interquartile range (IQR). (**D**–**H**) For endpoint studies, females were challenged subcutaneously in dorsal foot skin with 750 PFU ZIKV and mosquito saliva at day 28 and monitored for clinical signs. (**D**) Survival was plotted using a Kaplan–Meier curve (Kaplan–Meier, log rank (Mantel–Cox) with post hoc Holm–Sidak’s multiple comparisons test). The pink, green and black asterisks in the survival graph represent the *p*-value of the difference between Placebo and adjuvanted SAL vaccinated group (pink), placebo and VLP (green) and placebo and VLP+SAL (black). (**E**) Body weight post-infection was recorded, presented as percentage change from baseline (day of challenge). Line graph represents group medians + IQR. Black crosses (+) represent days on which animals were culled. (**F**) Viral RNA was quantified by qPCR in the skin, (**G**) spleen and (**H**) brain, presented as copies of ZIKV env per 10^7^ copies of 18S (Kruskal–Wallis with Dunn’s correction). Triangles, dots, squares and inverted triangles represent individual mice and bars represent group medians + IQR. Dotted lines, where plotted, represent the assay limits of detection. * = *p* ≤ 0.05, ** = *p* ≤ 0.01.

Similar to the observations in the CHIKV model, analysis of viraemia using the TCID50 assay showed that, at 2 dpi, median infectious-virus titres were close to the assay limit in the ZIKV VLP and ZIKV VLP + SAL groups, although detectable titres remained in individual animals receiving ZIKV VLPs alone (Figure 3A). In contrast, substantially higher titres were observed in the sera of placebo mice and mice immunised with SAL alone. At the same timepoint, qPCR to detect viral RNA showed that both ZIKV VLP and ZIKV VLP + SAL immunisation significantly reduced viral RNA copies compared to placebo in the spleen (Figure 3B), consistent with reduced systemic dissemination. At 4 dpi, there were no longer notable differences between groups in the serum and spleen (Supplementary Figure S1). Viral RNA was quantified in the brain at 4 dpi, when the virus is expected to be detectable, making CNS viral burden a key determinant of disease progression. High quantities of viral RNA were detected in the brains of mice immunised with placebo or SAL alone. At 4 dpi, brain viral RNA was reduced in both VLP-containing groups compared with the placebo and SAL groups, with statistically significant differences, as indicated in Figure 3C. The median viral RNA level was lowest in mice immunised with ZIKV VLP + SAL, although this did not differ significantly from ZIKV VLP alone.

In a separate study, mice were monitored for clinical signs after ZIKV infection for up to 15 days. Clinical monitoring revealed the clearest differences between groups (Figure 3D,E). Here, only mice receiving ZIKV VLP + SAL had significantly improved survival compared with all other groups (75%, *p* ≤ 0.05) (Figure 3D). These mice also lost less weight, and some began to regain weight, whereas all other groups continued to decline without recovery (Figure 3E). At the clinical endpoint of each mouse, analysis of tissue viral RNA showed significantly reduced ZIKV RNA in the skin (Figure 3F) at the inoculation site in mice immunised with ZIKV VLP + SAL, compared with placebo and ZIKV VLP alone. In the spleen (Figure 3G), ZIKV VLP immunisation significantly reduced viral RNA compared with the placebo, whereas viral RNA in the ZIKV VLP + SAL group was not significantly different from the placebo. In the brain (Figure 3H), ZIKV VLP vaccination alone did not significantly reduce viral RNA compared with the placebo, whereas ZIKV VLP + SAL immunisation significantly reduced brain viral RNA compared with both placebo and ZIKV VLP alone.

Collectively, these data indicate that the combined ZIKV VLP + SAL vaccination reduced viral RNA at the inoculation site and in the brain, improved survival, and enhanced the protective effect of the ZIKV VLP vaccination alone.

## 4. Discussion

In this study, we present the design of a novel vaccine, SAL, comprising conserved peptides from the saliva of female *Aedes* and *Culex* mosquitoes, and we evaluate its efficacy in two models of arboviral disease, CHIKV and ZIKV.

SAL administered alone did not provide any detectable protection in either model. However, when administered together with commercial ZIKV or CHIKV VLPs, SAL enhanced the protective effect of the VLPs in both models. The efficacy studies in the ZIKV and CHIKV animal models were conducted independently at separate institutions, and investigators were blinded to treatment groups until data analysis was completed.

Combining SAL with VLPs reduced viral load in the ipsilateral ankle and contralateral ankle of mice challenged with CHIKV, and reduced skin and brain viral RNA in mice challenged with ZIKV, consistent with reduced systemic dissemination and/or reduced seeding of distal tissues. Although the mechanism by which SAL enhances VLP-mediated protection remains to be defined, the data are most consistent with improved control at, or soon after, the inoculation site. Mosquito saliva acts primarily within the skin, where it can alter vascular permeability, recruit virus-permissive myeloid cells and reshape local innate immune responses, thereby promoting early arbovirus replication and dissemination [26]. Several SAL peptides contained in the SAL vaccine formulation are derived from salivary protein families with known or proposed roles in regulating barrier function and inflammatory recruitment. For example, SAL-42 and SAL-10 originate from the serine protease/CLIPA3/phenoloxidase-activating factor family of salivary proteins, which have been implicated in endothelial barrier disruption [27]. SAL-43 is derived from the *Ae. aegypti* bacteria-responsive protein AgBR1, which has been shown to upregulate chemoattractive chemokines and promote neutrophil recruitment to the bite site [28]. In addition, *Ae. aegypti* bites and saliva induce an inflammatory influx of myeloid cells that can enhance infection with genetically distinct arboviruses, including ZIKV, Semliki Forest virus and Bunyamwera virus, by increasing the availability of virus-permissive cells at the inoculation site [16,25,29]. In this context, SAL-induced immunity may not act directly on the virus, but could modify the activation state, composition or antiviral capacity of cells recruited to the site of virus/saliva challenge.

This interpretation also provides a plausible explanation for the reduced ZIKV RNA detected in the brain after VLP + SAL vaccination. Rather than salivary proteins acting directly on the blood–brain barrier, reduced brain viral RNA may simply reflect improved early control of infection, leading to lower systemic viral burden and reduced probability of central nervous system (CNS) seeding. Consistent with this, VLP + SAL reduced viral RNA in the skin at the clinical endpoint and reduced brain viral RNA more effectively than VLPs alone. We therefore interpret the brain data as evidence of improved overall control of ZIKV dissemination.

Combining SAL with VLPs also reduced viral burden directly at the inoculation site, including the ipsilateral ankle of mice challenged with CHIKV and the skin of mice challenged with ZIKV. Mosquito saliva contains proteins that can suppress innate antiviral responses and promote a local environment favourable to viral replication [26]. Peptide SAL-3 is derived from adenosine deaminase, which has previously been shown to inhibit IFN-α and IFN-β mRNA expression in DENV-infected keratinocytes [30]. Salivary proteins may also modulate adaptive immunity by altering the kinetics and distribution of T- and B-cells, and may polarise host responses from Th1 to Th2, although this effect varies between viral animal models [15,31,32,33]. By targeting immunomodulatory salivary proteins, SAL vaccination may therefore alter the recalled immune response to saliva upon challenge, favouring a local environment that is more supportive of antiviral control. Alternatively, SAL may mediate its effects through bystander activation of adaptive immune responses that enhance responses to the co-administered VLP and, subsequently, to the viral challenge. Consistent with this possibility, recent transcriptomic analysis of skin biopsies from human volunteers after *Ae. aegypti* or *Ae. albopictus* mosquito biting showed that vaccination with another salivary protein-based vaccine, AGS-v PLUS, enhanced Th1- and CD8+ T-cell-associated gene expression and suppressed pathways associated with neutrophilic inflammation and epithelial stress compared with the placebo, together suggesting enhanced antiviral capacity [19].

When vaccinated mice were challenged with either CHIKV or ZIKV, SAL alone did not confer protection despite its immunogenicity. This suggests that, under the dosing and challenge conditions assessed here, anti-saliva immunity was not sufficient by itself to control infection but could enhance protection when combined with virus-directed VLP immunity. Other mosquito saliva vaccines, such as AGS-v PLUS [18] and AgTRIO [34], have demonstrated moderate efficacy, albeit in a murine malaria model. It is possible that alternative dosing, timing, formulation, or delivery approaches could increase the magnitude, localisation, or quality of the SAL-induced response and improve efficacy. One future goal would be to combine SAL and VLPs into a single vaccine formulation without immune interference, reducing the number of injections required. While peptides are generally amenable to lyophilisation and room-temperature storage, VLPs are typically refrigerated; however, new advances may make room-temperature storage of both components possible [35,36]. These factors are important for vaccines intended for use in LMICs, where cold-chain capacity and access to healthcare facilities may be limited.

Further optimisation of SAL composition may also improve efficacy. Vector saliva is a complex mixture, and individual components can have divergent effects on infection; a few have been reported to enhance antiviral protection, while most promote infection [13]. This was exemplified by a sand fly saliva vaccine, where an isolated salivary protein enhanced survival in a leishmaniasis model, whereas the complex saliva mix did not provide equivalent protection [37]. It is therefore possible that some SAL components are immunogenic but not protective, or that protective responses could be improved by refining the peptide composition. Moreover, immunogenicity of the peptides could be enhanced by optimising the vaccine formulation. In this proof-of-concept study, the adjuvant Montanide ISA-51 was chosen based on previous clinical data obtained using this adjuvant with AGS-v and AGS-v PLUS, two peptide-based mosquito salivary antigen vaccines developed using the same platform as SAL [17,18]. In these studies, ISA-51-adjuvanted peptides administered subcutaneously demonstrated superior induction of T- and B-cell responses compared to non-adjuvanted peptides and peptides formulated with an Alum-based adjuvant. Further studies could be conducted to optimise the adjuvant and determine how the different vaccine-specific immune responses affect efficacy.

We focused on CHIKV and ZIKV because both are transmitted by *Aedes* mosquitoes, represent two genetically distinct families of viruses that impose an increasing global burden, and have an unmet need for safe, effective, and accessible interventions. For future development, it will also be important to determine whether SAL alters the quality, durability, or safety of virus-directed antibody responses. In particular, antibody-dependent enhancement is an important consideration for flavivirus vaccine development, especially in dengue-relevant settings [38,39]. Mosquito saliva extract has also been shown to enhance antibody-dependent enhancement (ADE) in dengue infection [40]. Future studies should therefore assess whether combining SAL with virus-directed vaccines affects ADE-relevant outcomes, particularly for flaviviruses where pre-existing cross-reactive antibodies may influence disease.

A further limitation is that only a single VLP dose was tested. The study was designed as a proof-of-concept comparison of VLP vaccination with or without SAL, using the same VLP dose in both groups, rather than as a VLP dose-optimisation study. The 0.5 µg dose was deliberately chosen to be partially, rather than fully, protective so that any additive contribution of SAL would be detectable, but it was not defined by dose-ranging in our own models. Therefore, while the data show that addition of SAL improved protection under the conditions tested, they do not establish whether this effect would be maintained across a broader VLP dose range or whether SAL would have the greatest benefit at submaximal, intermediate, or higher VLP doses.

In addition, VLP-specific antibody responses were not measured in this study. The immunogenicity analyses focused on responses to the salivary peptide component, whereas the efficacy experiments assessed virological and clinical outcomes after challenge. We therefore cannot determine from the present data whether SAL enhanced protection by altering virus-specific humoral immunity, by modifying the recalled response to mosquito saliva at the inoculation site, or through another mechanism. Future studies should measure VLP-specific binding and neutralising antibody responses, and assess whether SAL changes their magnitude, quality, durability, or protective functions.

A limitation is that saliva input was standardized by mosquito equivalent rather than by measuring total salivary protein concentration, as per published studies [16]. Although pooled saliva was distributed equivalently across vaccine groups within each challenge experiment, total protein content and batch-to-batch variation were not directly quantified.

Translation to human application will require careful consideration of target populations. For CHIKV, safety concerns with licensed vaccines in older adults highlight the importance of confirming the safety and efficacy of CHIKV VLP + SAL vaccination in older populations, while extension to younger age groups may also be valuable. For ZIKV, the target population would include individuals of reproductive potential, ideally before pregnancy, making it essential to assess vaccine safety in females of different age groups and to determine the effect of vaccination on pregnancy and foetal health. A limitation of the present study is that ZIKV fixed-timepoint and clinical-monitoring experiments were conducted as independent, internally sex-matched cohorts. This reflected availability from the *Ifnar1*-deficient breeding colony and 3R considerations but means that no direct comparison can be made between male- and female-derived datasets. In addition, a limitation of the CHIKV efficacy study is that it was performed in female mice only. Future studies should assess whether SAL + VLP vaccination provides comparable protection in male and female animals, and whether biological sex influences vaccine-induced protection or disease outcome. Overall, the clearer effect of VLP + SAL in the clinical endpoint study could therefore reflect the later timepoint, biological sex, or technical differences in inoculation method. Future studies should address these variables directly using matched experimental designs.

Finally, SAL was designed with broad protection in mind. Extending this research to other arboviruses transmitted by *Aedes* and *Culex* mosquitoes, such as dengue virus and West Nile virus, will be important to determine the breadth of protection that SAL can offer. It will also be useful to combine SAL with other virus-directed vaccine platforms beyond VLPs to better define its mechanism of action and translational potential. For example, comparing SAL in combination with CHIKV VLPs versus live-attenuated CHIKV vaccines may help determine whether SAL preferentially enhances particular types of virus-directed immunity.

## 5. Conclusions

Together, the data presented show that the protection offered by VLP vaccines against arboviruses can be enhanced by the addition of a vaccine component targeting mosquito salivary antigens. Further work is now needed to define the mechanism of action, optimise the SAL composition and formulation, and determine whether this approach can provide broader protection against mosquito-borne viral diseases.

## Figures and Tables

**Table 1:** List of primers, primer orientations, sequences, and NCBI references for all qPCR primers used in this study.

Gene Name	Orientation	Sequence	NCBI Reference
18S	Forward	gactcaacacgggaaacctc	NR_003278. 1
	Reverse	taaccagacaaatcgctccac	
18S Standard	Forward	cgtagttccgaccataaacga	NR_003278. 1
	Reverse	acatctaagggcatcacagacc	
ZIKV ENV	Forward	ggaggctgagatggatggtg	KX_197192.1
	Reverse	cagtgtttcagccgggatct	
ZIKV ENV Standard	Forward	aggcaaactgtcgtggttct	KX_197192.1
	Reverse	tcagacccaaccacatcagc	
CHIKV Nsp1	Forward	ccgactcaaccatcctggat	FJ_959103.1
	Reverse	ggcagacgcagtggtacttcct	

**Table 2:** Mosquito salivary proteins from which SAL peptides were derived.

Peptide	Protein of Origin	Mosquito Genus	Length (aa)	GenBank Reference
SAL-3	Adenosine deaminase	*Aedes*	26	AAL76033.1
SAL-8	Glutathione S transferase D7	*Aedes* and *Culex*	23	XP_001658010.2 XP_039443046.1
SAL-10	Serine protease/CLIPA3/Phenoloxidase activating factor 5	*Culex*	22	EDS43263.1
SAL-21	29 kDa salivary protein	*Culex*	25	EDS39796.1
SAL-27	possibly secreted protein	*Culex*	26	EDS34868.1
SAL-33	62 kDa putative secreted protein (3)	*Aedes*	28	AAV90681.1
SAL-38	chorion peroxidase	*Culex*	26	XP_039430972.2
SAL-42	Serine protease/CLIPA3/Phenoloxidase activating factor 7	*Culex*	22	EDS43263.1
SAL-43	*Ae. aegypti* bacteria responsive element	*Aedes*	24	ABF18180.1

## Data Availability

Archives of the data collected are not publicly available.

## Supplementary Materials

The following supporting information can be downloaded at: https://www.mdpi.com/article/10.3390/vaccines14080723/s1, Supplementary Table S1 Part 1. Summary of protocol for animal studies: mice & vaccinations; Supplementary Table S1 Part 2. Summary of protocol for animal studies: viral challenge & endpoints; Supplementary Table S2. Salivary peptide selection panel; Supplementary Figure S1. Quantification of ZIKV in tissues over time.
