# Supplementary material for: Mosquito Saliva Subunit Vaccine Enhances Efficacy of VLP Vaccines Against Zika and Chikungunya Viruses
Source: Vaccines (Basel). 2026 Aug 21;14(8):723. doi: 10.3390/vaccines14080723 (PMC13517861; doi:10.3390/vaccines14080723)
Supplement: Supplementary file 1 [file vaccines-14-00723-s001.zip › vaccines-4470169-supplementary.pdf]

Study	Mouse strain	Sex	Age at first vaccination	Groups/ formulations	n per group	Immunisation schedule
Salivary peptide immunogenicity screening	C57BL/6J	Female	7-9 weeks	Placebo or 5-peptide pools containing 10 nmol of each peptide, formulated in Montanide ISA-51	n=4	Subcutaneous prime and boost, day 0 and day 14
Final SAL immunogenicity study	C57BL/6J	3 male + 3 female per group	7-9 weeks	Placebo or final SAL formulation containing nine salivary peptides, 10 nmol of each peptide, formulated in Montanide ISA-51	n=6	Subcutaneous prime and boost, day 0 and day 14
CHIKV efficacy study	C57BL/6	Female	4 weeks	Placebo; SAL; CHIKV VLP; CHIKV VLP + SAL.SAL contained 10 nmol of each peptide. VLP dose was 0.5 µg. Formulations were adjuvanted with Montanide ISA-51; in the combination group, SAL and VLP were administered on opposite flanks	n=8 per group per timepoint	Subcutaneous prime and boost, day 0 and day 14
ZIKV fixed-timepoint efficacy study	Ifnarl-/- on C57BL/6 background	Male	6-12 weeks	Placebo; SAL; ZIKV VLP; ZIKV VLP +SAL.SAL contained 10 nmol of each peptide. VLP dose was 0.5 µg. Formulations were adjuvanted with Montanide ISA-51; in the combination group, SAL and VLP were administered on opposite flanks.	n=8 per group per timepoint; n=16 per group across both fixed timepoints	Subcutaneous prime and boost, day 0 and day 14
ZIKV clinical-sign/ survival study	Ifnarl-/- on C57BL/6 background	Female	6-12 weeks	Placebo; SAL; ZIKV VLP; ZIKV VLP + SAL.SAL contained 10 nmol of each peptide. VLP dose was 0.5 µg. Formulations were adjuvanted with Montanide ISA-51; in the combination group, SAL and VLP were administered on opposite flanks	n=8	Subcutaneous prime and boost, day 0 and day 14

Supplementary Table S1 Part 1. Summary of protocol for animal studies: mice & vaccinations.

Study	Challenge	Saliva input	Sampling / endpoint
Salivary peptide immunogenicity screening	None	None	Euthanised day 28; spleen and serum collected for FluoroSpot and ELISA
Final SAL immunogenicity study	None	None	Euthanised day 28; spleen and serum collected for FluoroSpot and ELISA
CHIKV efficacy study	Day 28: subcutaneous challenge into the left hind footpad with 10^6 PFU CHIKV plus mosquito saliva in a total volume of 20 µL	Pooled *Ae. aegypti* saliva. Each mouse received the equivalent of saliva from two mosquito probing/salivation events. The pooled saliva preparation was used so that mice received equivalent saliva input across vaccine groups	Body weight and foot swelling monitored daily. Mice euthanised at 2 dpi or 7 dpi. Blood, ipsilateral ankle and contralateral ankle collected for infectious virus and/or viral RNA quantification
ZIKV fixed-timepoint efficacy study	Day 28: subcutaneous challenge into the hind footpad with 750 PFU ZIKV plus mosquito saliva in a total volume of 20 µL	Pooled *Ae. aegypti* saliva. Each mouse received the equivalent of saliva from two mosquito probing/salivation events. The pooled saliva preparation was used so that mice received equivalent saliva input across vaccine groups	At 2 dpi, n=8 per group euthanised; spleen, popliteal lymph node, inoculation-site skin and serum collected. At 4 dpi, n=8 per group euthanised; skin, spleen, half brain and serum collected
ZIKV clinical-sign/ survival study	Day 28: subcutaneous challenge into dorsal left foot skin with 750 PFU ZIKV plus mosquito saliva in a total volume of 4 µL using 33-gauge Hamilton microneedles	Pooled *Ae. aegypti* saliva. Each mouse received the equivalent of saliva from two mosquito probing/salivation events. The pooled saliva preparation was used so that mice received equivalent saliva input across vaccine groups	Mice monitored three times daily for up to 15 days. Animals were humanely culled if they reached≥15% body-weight loss or predefined clinical signs. Skin, spleen and brain collected at clinical endpoint

Supplementary Table S1 Part 2. Summary of protocol for animal studies: viral challenge & endpoints.

Peptide	Total Ig Vaccine	Total Ig Placebo	IFNy Vaccine	IFNy Placebo	IL-2 Vaccine	IL-2 Placebo	TNFa Vaccine	TNFa Placebo
SAL-1	0.05	0.03	6	1	40 *	1	0	0
SAL-2	0.09	0.05	12 *	0	93 *	0	0	0
SAL-3	2.76 *	0.15	31 *	0	158 *	0	0	0
SAL-4	0.16	0.14	0	1	1	0	0	0
SAL-5	0.49	0.20	0	1	3	0	0	0
SAL-7	0.13	0.09	0	0	1	0	0	0
SAL-8	0.27	0.14	1	28	28 *	0	0	0
SAL-9	1.00 *	0.03	0	1	4	0	0	0
SAL-10	0.56	0.05	1	13	26 *	0	1	0
SAL-11	0.29 *	0.07	0	0	0	0	0	0
SAL-12	0.20	0.22	23	33	2	0	0	0
SAL-13	0.05	0.05	16	0	38 *	0	1	0
SAL-14	0.05	0.05	1	15	1	0	0	0
SAL-15	0.08	0.08	0 *	27	1	0	0	0
SAL-16	0.06	0.03	1	0	2	0	0	0
SAL-17	0.26	0.25	26 *	0	1	1	0	0
SAL-18	0.10	0.03	26 *	0	4	1	0	0
SAL-19	0.09	0.07	71 *	0	8	1	0	0
SAL-20	0.15	0.15	3	0	1	0	0	0
SAL-21	1.09 *	0.12	23 *	0	39	3	1	0
SAL-22	0.03	0.03	8	6	8	4	1	1
SAL-23	0.25	0.32	8	0	25	13	1	0
SAL-24	0.04	0.04	40	2	49	3	6	0
SAL-25	0.02	0.02	0	4	8	13	1	0
SAL-26	0.04	0.03	9	26	19	12	6	1
SAL-27	0.32	0.25	398 *	3	271 *	18	58	4
SAL-28	0.12	0.12	11	4	11	19	0	2
SAL-29	0.25	0.16	5	0	6	15	1	1
SAL-30	0.46	0.29	0	0	23	18	0	1
SAL-32	0.03	0.03	5	0	28	16	1	1
SAL-33	0.01	0.02	229	6	221 *	9	19 *	0
SAL-34	0.48 *	0.12	5	8	33	10	1	0
SAL-35	0.28	0.04	13	0	19	1	3	2
SAL-36	0.77 *	0.05	3	0	16	11	1	3
SAL-37	0.36	0.07	6	3	14	8	1	1
SAL-38	0.78 *	0	112 *	0	94 *	11	11	1
SAL-39	1.10	0.03	11	10	11	18	3	1
SAL-40	0.03	0.05	14	14	18	16	3	1
SAL-42	0.04	0.05	526	13	94 *	13	16	3
SAL-43	3.14 *	0.01	261 *	1	197 *	24	34 *	2

Median A_{450}-A_{570}: 0 1 2 3

Median SFU/10^6 cells: 0 200 400

Supplementary Table S2. Salivary peptide selection panel. 40 salivary peptides were selected *in silico,* manufactured as synthetic peptides and pooled in groups of 5 peptides/group. Female C57BL/6 mice were subcutaneously injected on day 0 and 14 with saline (placebo) or a 10nmol equimolar mix of a 5-peptide pool in Montanide ISA-51 adjuvant, then euthanised on day 28 (n = 4). Total serum immunoglobulin (Ig) against each peptide was determined by ELISA, presented as absorbance normalised to background ($A_{450-570nm}$). IFNγ, IL-2 and TNFα cytokine responses were assessed by FluoroSpot in splenocytes re-stimulated with the peptides, presented as spot forming units (SFU) per million cells, normalised to unstimulated cell controls. P-value was calculated by Mann Whitney U test,* =$p \leq 0.05$.

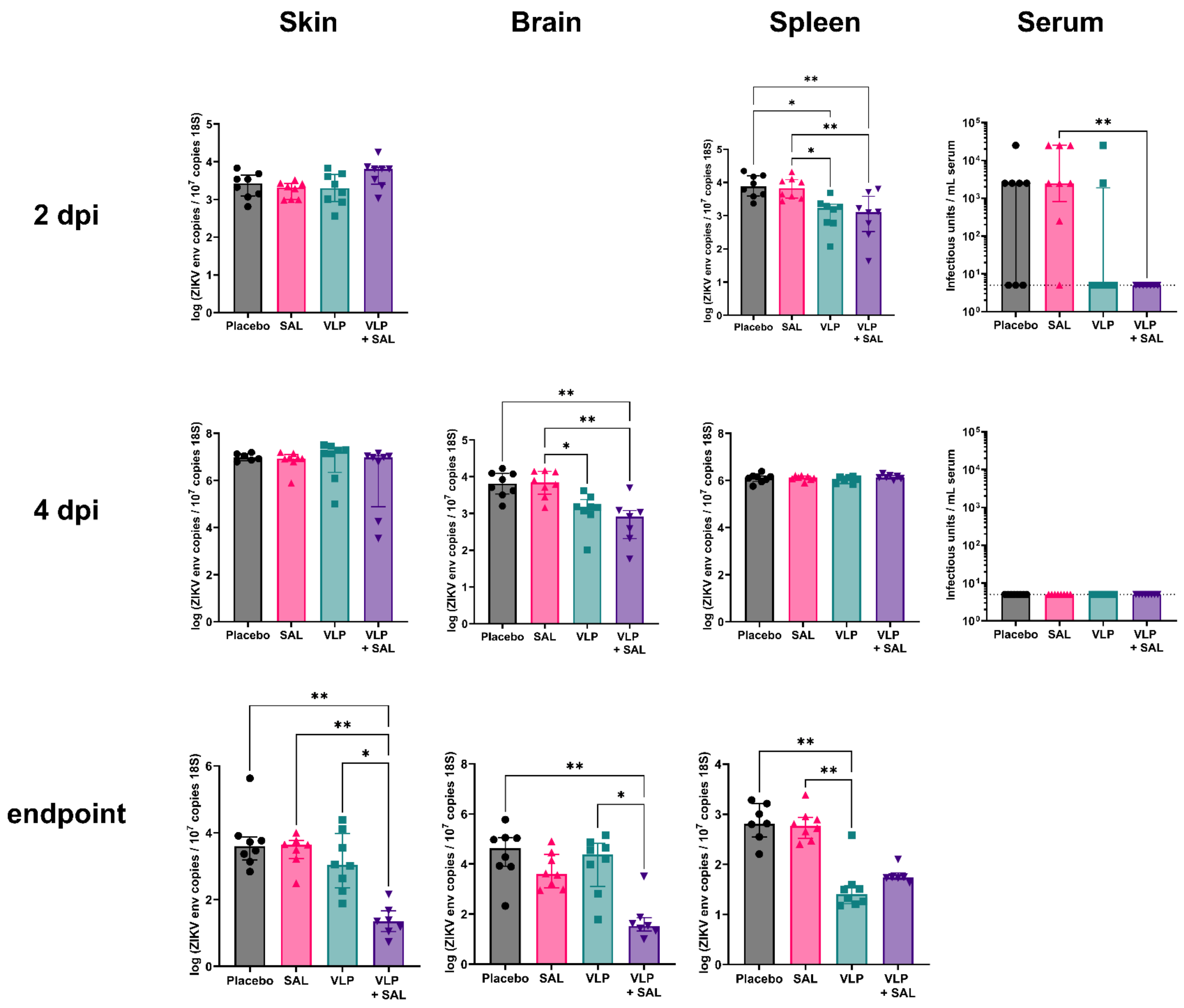

Supplementary Figure S1. Quantification of ZIKV in tissues over time. Ifnarl-/- mice were subcutaneously injected on day 0 and 14 with 100 µL of adjuvanted saline (placebo), adjuvanted SAL vaccine (10 nmol of each peptide), adjuvanted ZIKV VLPs (0.5µg), or both adjuvanted SAL and VLPs in opposite flanks. For fixed timepoint studies, male mice were challenged subcutaneously in the footpad with 750 PFU ZIKV and mosquito saliva at day 28. Half the mice were euthanised 2 days post-infection (dpi) and the other half were euthanised 4 dpi (total n=8 per group, per timepoint). For endpoint studies, female mice were challenged subcutaneously in the footpad with 750 PFU ZIKV and mosquito saliva on day 28 and monitored until day 12, or until clinical endpoints were reached. Infectious virus in serum was quantified by viral titration in Vero cells, presented as infectious virus per mL serum collected. Viral RNA was quantified in the spleen, skin and brain by qPCR, presented as copies of ZIKV envelope (env) RNA per 10^7 copies of housekeeping gene 18S. Points represent individual mice and bars represent median with interquartile range. Kruskal-Wallis test with Dunn's correction was performed between each vaccine group and the placebo group. * = p ≤0.05, ** = p ≤0.01.
